# Brentuximab vedotin for relapsed or refractory Hodgkin lymphoma: experience in Turkey

**DOI:** 10.1007/s00277-014-2215-9

**Published:** 2014-09-18

**Authors:** A. Salihoglu, T. Elverdi, I. Karadogan, S. Paydas, E. Ozdemir, G. Erdem, N. Karadurmus, G. Akyol, L. Kaynar, ZA Yegin, G. Sucak, V. Ozkocaman, P. Topcuoglu, M. Ozcan, E. Birtas, H. Goker, Z. Baslar, B. Ferhanoglu

**Affiliations:** 1 Cerrahpasa Medical Faculty, Department of Internal Medicine, Division of Hematology, Istanbul University, Istanbul, Turkey; 2 Division of Hematology, Antalya Medstar Hospital, Antalya, Turkey; 3 Department of Internal Medicine, Division of Medical Oncology, Cukurova University, Adana, Turkey; 4 Institute of Oncology, Stem Cell Transplantation Unit, Hacettepe University, Ankara, Turkey; 5 Department of Internal Medicine, Gulhane Military Medical Academy, Division of Medical Oncology, Ankara, Turkey; 6 Erciyes University, Department of Internal Medicine, Division of Hematology, Kayseri, Turkey; 7 Department of Internal Medicine, Division of Hematology, Gazi University, Ankara, Turkey; 8 Department of Internal Medicine, Division of Hematology, Uludag University, Bursa, Turkey; 9 Department of Internal Medicine, Division of Hematology, Ankara University, Ankara, Turkey; 10 Kocaeli University, Department of Internal Medicine, Division of Hematology, Kocaeli, Turkey; 11 Hacettepe University, Department of Internal Medicine, Division of Hematology, Ankara, Turkey; 12 Koc University, Department of Internal Medicine, Division of Hematology, Istanbul, Turkey

**Keywords:** Brentuximab vedotin, Hodgkin Lymphoma, Relapsed, Refractory

## Abstract

Current treatment modalities can cure up to 70–80 % of patients with classical Hodgkin lymphoma. Approximately, 20–30 % of patients require further treatment options. Brentuximab vedotin has been approved for the treatment of relapsed and refractory Hodgkin lymphoma. In the present study, we report the experience with brentuximab vedotin as single agent in 58 patients with relapsed or refractory Hodgkin lymphoma. The objective response rate was 63.5 % with 13 complete responders (26.5 %) among 49 patients evaluated at the early phase of treatment (2–5 cycles). Upon treatment prolongation (≥6 cycles), 37 patients achieved a final objective response rate of 32.4 % with 21.6 % of complete and 10.8 % of partial response. Overall survival at 12 months was 70.6 %, and progression-free survival at 12 months was 32.8 %. Median overall survival could not be reached and median progression-free survival was 7 months. While the median duration of response was 9 months in the whole cohort, it was 11.5 months in the complete responders. Complete response rates in patients treated with >3 chemotherapy regimens before brentuximab vedotin were significantly lower (*p* = 0.016). Fourteen patients were subsequently transplanted. In conclusion, brentuximab vedotin provided a bridge to transplantation in approximately one quarter of the patients. The declining response rates during the course of treatment suggest that transplantation should be implemented early during brentuximab vedotin treatment.

## Introduction

Classical Hodgkin lymphoma (cHL) is one of the most curable cancers. However, approximately a third of patients will relapse after first-line therapy and 15 % will fail both first and second line therapies, and therapeutic options are still quite limited in these patients [1]. Brentuximab vedotin (BV) is a novel antibody-drug conjugate composed of a CD30-directed recombinant chimeric immunoglobulin G1 covalently linked to a synthetic antitubulin chemotherapeutic agent monomethyl auristatin E. BV showed an impressive activity against relapsed/refractory cHL [2].

We would like to provide more information on the efficacy and safety of BV and contribute to the determination of the best role and timing of BV treatment in the current study.

## Design and methods

BV is approved for the treatment of cHL after failure of autologous transplantation or after failure of at least two prior multi-agent chemotherapy regimens in patients that are not candidates for transplantation. Eleven Turkish institutions participated in this multicenter, retrospective study. Eligible patients were required to be treated with at least two courses of BV and to have available clinical documentation and staging investigations. Inclusion of the patients with organ dysfunction was based on the decision of the attending physician.

All patients underwent baseline assessments including physical examination, routine laboratory tests, and radiological examinations [computed tomography (CT), positron emission tomography (PET/CT)] prior to the treatment. Patients were included without any limitations regarding performance status and organ function. All of the patients provided written informed consent.

BV is imported via the named-patient-program. BV was given at a dose of 1.8 mg/kg intravenous infusion over 30 min every 3 weeks in an outpatient setting. Since the drug is received institutionally, the relative dose intensity was calculated as total chemotherapy dose delivered/total chemotherapy duration [3].

The primary endpoint of the study was the objective response rate (ORR); secondary endpoints were safety, overall survival (OS), and progression-free survival (PFS). Response was assessed by PET/CT or CT early during disease course after 2–5 cycles [4] and after ≥6 cycles using the international working group revised response criteria [5].

Safety and tolerability were assessed before each BV cycle according to National Cancer Institute Common Terminology Criteria for Adverse Events Version 4.0. For grade 3 toxicity, dose reduction of BV to 1.2 mg/kg was recommended.

OS was defined as the time from the initiation of BV to death of any cause. PFS was the time from treatment initiation to progression, relapse, or death of any cause [5]. Primary refractory disease was defined as no complete remission or relapse within 3 months of first line therapy [6]. Both OS and PFS were censored at the date of last information. Median duration of response, PFS, and OS rates along with two-sided 95 % confidence interval (CI) was estimated using the Kaplan-Meier method. Survival functions were compared using log-rank test. Analysis of the data was carried out using STATA 11.1 SE software.

## Results

From March 2011 to July 2013, 58 patients in eleven Turkish centers were treated with BV within the named patient program. Outcomes and safety/toxicity data of 58 patients with relapsed or refractory cHL were analyzed in this study, aiming to evaluate the efficacy and safety of BV.

Demographics and disease characteristics of the patients are summarized in Table 1. The median age at diagnosis was 26 years, and 64 % were males. Nodular sclerosis was the most frequent (79 %) histologic subtype, half of the patients (49 %) had primary refractory disease, and 72 % were refractory to the last treatment given before BV. Most of the patients (77.5 %) had advanced stage disease and approximately half of them (47 %) had B-symptoms.

**Table 1:** Patient demographics and disease characteristics (*N* = 58)

Median age at diagnosis, years (range)			26 (13–62)
Sex, male/female, *n*/*n*			37/21
			*n* (%)
Systemic symptoms at diagnosis^a^			46 (82)
Histological subtype	Nodular sclerosis		46 (79)
	Mixed cellularity		6 (10)
	Lymphocyte rich		1 (2)
	Not determined		5 (9)
Frontline Hodgkin lymphoma regimen ABVD			57 (98)
Prior radiation therapy			45 (77.5)
Prior transplantation (*N* = 49)^b^	autologous		39 (79.6)
	allogeneic		1 (2.05)
	autologous + allogeneic		8 (16.3)
	autologous + haploidentical allogeneic		1 (2.05)
Median prior chemotherapy regimens			4 (2–7)
Primary refractory disease to frontline therapy			28 (49)
Refractoriness to most recent therapy			42/58 (72)
Disease status at the initiation of brentuximab vedotin	Stage ΙΙΙ/ΙV		45 (77.5)
	B-symptoms		27 (47)
	Performance status	ECOG score 0	14 (24)
		ECOG score 1	32 (55)
		ECOG score 2	12 (21)

*ABVD* adriamycin + bleomycin + vinblastine + dacarbazine, *ECOG* Eastern Cooperative Oncology Group

^a^Two patients did not have systemic symptom evaluation results

^b^Nine patients (15 %) had not undergone stem cell transplantation previously because of progressive disease (PD) in seven, poor mobilization in one, and decision of the attending physician in another

**Fig 1:**
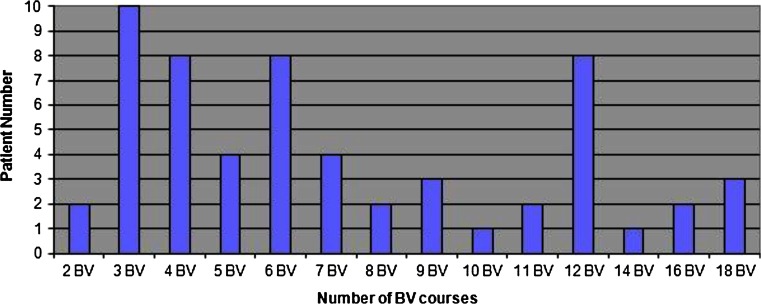
Number of brentuximab vedotin course

**Fig 2:**
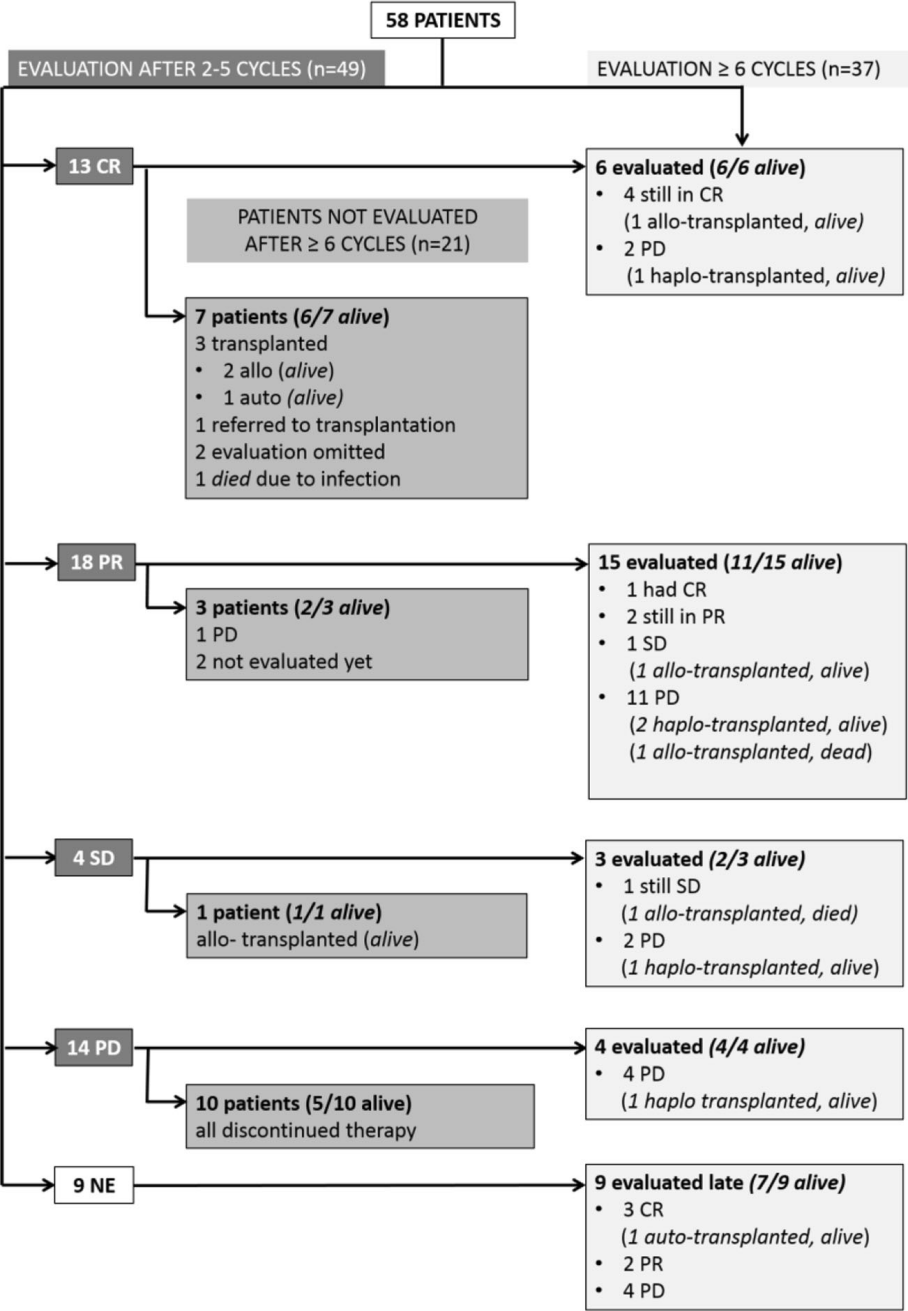
Results of post-BV assessments

A median of 7 (range, 2–18) courses of BV were given as a single agent (Fig. 1) and relative dose intensity was calculated as 81.6 %. The results of post-BV assessments are shown in Fig. 2. PET/CT was performed in 36 patients and 13 were assessed with CT early during treatment course. Thirty-one patients underwent PET/CT and 6 were evaluated with CT scan after ≥6 cycles of BV.

### Early assessment after 2–5 cycles of BV

After 2–5 cycles of BV, an objective response rate (ORR) of 63.5 % (*n* = 31) with 26.5 % (*n* = 13) CR was achieved.

In the subgroup of patients with an objective response, the median PFS duration was 9 months and PFS at 12 months was 44.34 % (95 % CI, 24.6 to 62.5 %). In the remaining 18 patients without an objective response, PFS was significantly lower (*p* < 0.001) with a median PFS of 3 months. OS at 12 months was 80.5 % (95 % CI, 59.0 to 91.5 %) and 57.9 % (95 % CI, 28.6 to 78.8 %) in patients with or without objective response, respectively. The median OS has not been reached in either group.

Among the 13 patients who achieved CR at the early evaluation after 2–5 treatment cycles, the frontline treatment regimen was ABVD (adriamycin + bleomycin + vinblastine + dacarbazine) and the median number of BV courses was nine. Other characteristics of these patients are given in Table 2. Of these 13 patients, one died due to infection; five underwent transplantation (three allogeneic, one haploidentical, and one autologous) after BV with a follow-up time of 1 month for two patients; 7, 8, and 12 months for three patients after transplantation; four could not find a matched donor and proceed to transplantation; one refused transplantation although there was a matched sibling donor; and one was preparing for allogeneic transplantation at the time of statistical analysis; one patient was not a transplant candidate, because allogeneic transplantation had been already performed.

**Table 2:** Characteristics of 13 patients who achieved complete remission at early evaluation, after 2–5 cycles of brentuximab vedotin

Median age at diagnosis, years (range)		25 (16–53)
Sex, male/female, *n*/*n*		9/4
Prior radiation therapy, *n* (%)		11 (85)
Prior transplantation, *n* (%)	autologous stem cell	9 (69)
	autologous + allogeneic	1 (7.7)
Number of previous chemotherapy regimens, median (range)		4 (2–6)
Refractory to frontline therapy, *n* (%)		3 (23)
Refractory to most recent therapy, *n* (%)		8 (61.5)

Five patients were referred to transplantation before 6 cycles of therapy. Three of them underwent allogeneic and one of them autologous stem cell transplantation. The fifth patient had not been transplanted yet at the time of statistical analysis.

Twenty-one patients did not undergo a later assessment of response after ≥6 cycles of treatment. Of these, 11 had disease progression during early treatment course and discontinued therapy, one died because of infection and two were awaiting response evaluation at the time of statistical analysis. Response assessment was omitted in two patients after achieving complete response (CR) during early response evaluation since these patients did not have any detectable clinical evidence of disease and disease-related symptoms. They were treated with 18 courses of BV but could not proceed with transplantation (one patient had no donor and the other had autologous and allogeneic transplantation before BV).

### Later assessment (after ≥6 cycles of BV)

Nine patients were not evaluated (NE) early during treatment course based on the attending physician’s decisions (three achieved CR, two achieved partial remission (PR), and four were found to have PD), but evaluated after ≥6 cycles.

**Fig 3:**
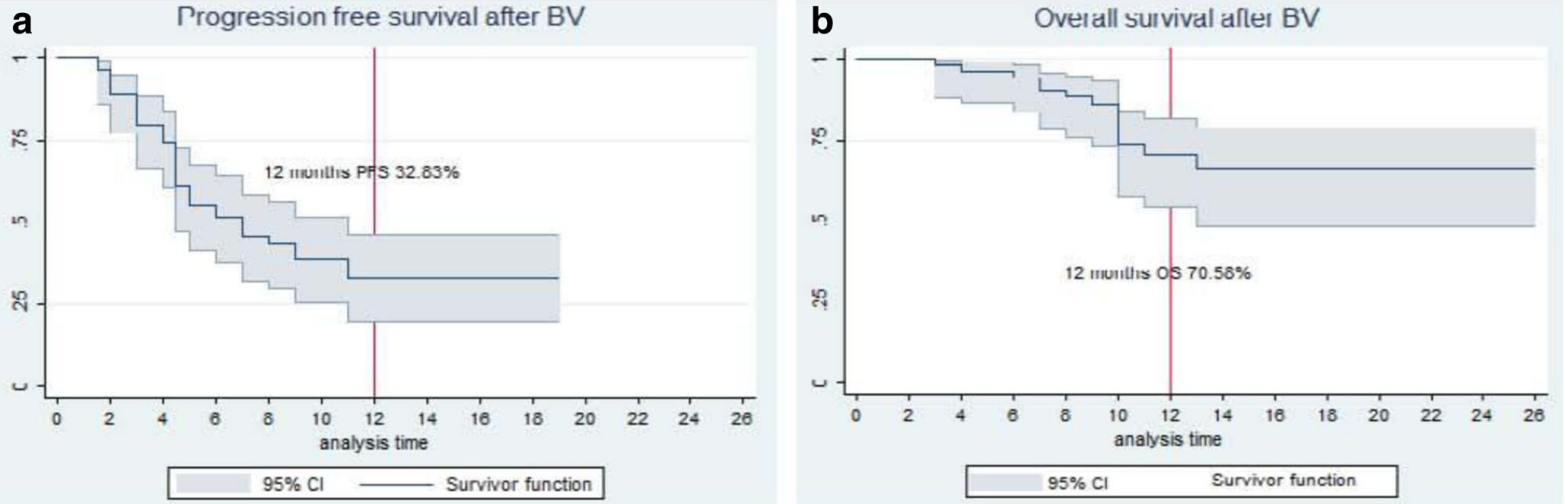
Kaplan-Meier plots and 95 % CIs for PFS and OS. **a** The median PFS was 7 months. **b** The OS at 12 months was 70.58 %. The median OS has not yet been reached

Response assessment after ≥6 cycles was performed with PET/CT in 31 of 37 patients and CT in 6 patients and showed an ORR of 32.4 % (*n* = 12) with CR in 8 patients (21.6 %). PFS at 12 months was 32.8 % (95 % CI, 19.7 to 46.6 %) and the median PFS duration was 7 months (95 % CI, 4.8 to 11.3 %) (Fig. 3a). Fourty-four patients were alive at the time of data analysis. OS at 12 months was 70.6 % (95 % CI, 54.4 to 81.9 %) and the median OS has not been reached yet (Fig. 3b).

Twelve patients had been treated with three or less previous chemotherapy regimens before BV and half of the patients achieved CR. CR rates in patients treated with >3 chemotherapy regimens before BV were significantly lower (15 %) than CR rates in patients with fewer prior therapies (*p* = 0.016).

Fourteen patients proceeded to transplantation (7 allogeneic, 5 haploidentical, and 2 autologous transplantation). Among these, 5 patients (36 %) entered transplantation in CR, while 3 (21 %) with stable disease (SD) and 6 (43 %) with PD (Table 3). After transplantation, 12 patients (85 %) survived. All five patients receiving transplantation in CR (3 allogeneic, 1 autologous, and 1 haploidentical) were in continuous CR after 1, 1,7, 8, and 12 months of transplantation, respectively. None of the patients entering transplantation with SD or PD (*n* = 9) could achieve remission after transplantation.

### Safety/toxicity

In general, the treatment was well tolerated with dose reduction required in five patients (three due to cytopenias, one peripheral neuropathy, one unknown). The most common (>10 %) treatment-related adverse events were fatigue, nausea, neuropathy, neutropenia, vomiting, myalgia, alopecia, and extremity pain (Table 4).

**Table 3:** Disease status at transplantation

	*N* (%)
Complete remission	5 (36)
Stable disease	3 (21)
Progressive disease	6 (43)

Neurological toxicity was observed in 20 patients: peripheral neuropathy pattern in 17, grade 3–4 neuropathy occurred in two, and oculomotor nerve palsy in one patient. Two patients from different centers suffered from generalized tonic convulsions, one of whom was on renal replacement therapy. Convulsions occurred after 5 and 2 cycles of BV treatment. Since drug-induced neurotoxicity could not be excluded, BV was stopped in both patients.

**Table 4:** Most common adverse events reported by >10 % of the patients regardless of relationship with brentuximab vedotin

Adverse event (AE)	Total number of patients with AE, *n* (%)	Grade 3–4 AE, *n* (%)
Fatigue	29 (50.0)	–
Nausea	19 (32.8)	2 (3.4)
Neuropathy	18 (31.0)	2 (3.4)
Neutropenia	16 (27.6)	1 (1.7)
Vomiting	15 (25.8)	1 (1.7)
Myalgia	15 (25.8)	2 (3.4)
Alopecia	12 (20.7)	–
Extremity pain	12 (20.7)	2 (3.4)
Pyrexia	8 (13.8)	–
Muscle spasm	8 (13.8)	–
Constipation	7 (12.0)	–
Pruritus	7 (12.0)	–

## Discussion

This multicenter, retrospective study was conducted to investigate activity and safety of BV in patients with cHL. Patients were managed in a non-trial setting, and the study population mainly consisted of heavily pretreated, high-risk patients. Although having multiply relapsed or refractory disease, ECOG performance status of the patients was ≤2.

In general, treatment was well tolerated, and toxicities were generally grade 1 and 2 in severity. Patients with organ dysfunction were not excluded. Namely, there were three patients with chronic kidney disease (one with stage 4 and two with stage 5, undergoing hemodialysis) included in the study. All three patients had received previous salvage chemotherapy including cisplatin. Two of these patients were treated without any dose modifications. One of these hemodialysis patients treated without dose reduction had generalized seizures after two cycles of BV and therapy was stopped. The other two patients with stage 4–5 chronic kidney disease did not experience any particular side effects or deterioration of kidney function during treatment course.

Interestingly, generalized seizures with a toxicity grade of 3 were reported in two patients from two different centers. One of the patients had stage 5 chronic kidney disease and seizures occurred after two cycles as mentioned previously. The other patient experienced seizures after 5 treatment cycles and treatment was stopped. Grade 3 oculomotor nerve disorder developed in one patient after 7 BV treatment cycles. Possible underlying causes were excluded in this patient. One patient with a previous diagnosis of demyelinating polyneuropathy did not experience any deterioration of neuropathy during the treatment course.

The retrospective nature of the analysis of data was a limitation of the present study.

The finding that patients with less prior chemotherapy regimens had better CR rates compared with more heavily pretreated patients (*p* = 0.016) should be interpreted cautiously because of the low number of less intensively treated patients and no data in the literature supporting this finding.

Similar to previous studies [6–9], our study also indicates that best responses to BV are observed after 2–5 cycles, early during the treatment course. Since best results are obtained with chemosensitive disease and minimal tumor burden both in autologous and allogeneic stem cell transplantation, transplantation should be considered earlier during BV treatment when best responses are achieved.

In the present study, nearly one quarter of patients (*n* = 14) underwent transplantation (7 allogeneic, 5 haploidentical and 2 autologous) after BV therapy, which is consistent with the current literature [8,10]. However, only 36 % of these patients (*n* = 5) were in complete remission before transplantation, and 43 % of patients (*n* = 6) were transplanted with chemoresistant PD. This poor pre-transplant disease control showed that BV was not used as an effective bridge to transplant in the present study. In spite of this fact, 85 % of the transplanted patients were alive. Consideration of transplantation early during BV treatment course can improve disease status at transplantation.
